# Waste Disposal Sites as All-You-Can Eat Buffets for Carrion Crows (*Corvus corone*)

**DOI:** 10.3390/ani9050215

**Published:** 2019-05-04

**Authors:** Doris Preininger, Bjoern Schoas, Diether Kramer, Markus Boeckle

**Affiliations:** 1 Vienna Zoo, 1130 Vienna, Austria; d.preininger@zoovienna.at; 2 Department of Landscape, Spatial and Infrastructure Sciences, University of Natural Resources and Life Sciences, 1190 Vienna, Austria; bjoern.schoas@gmail.com; 3 Steiermärkische Krankenanstaltengesellschaft m. b. H., 8010 Graz, Austria; diether.kramer@gmx.net; 4 Department of Cognitive Biology, University of Vienna, 1090 Vienna, Austria; 5 Department of Psychology, University of Cambridge, CB2 3EB Cambridge, UK

**Keywords:** abundance, anthropogenic food, *Corvus corone*, crow, corvid, ecology, waste management

## Abstract

**Simple Summary:**

Several bird species like common ravens, carrion crows, hooded crows, and rooks are held responsible for damage to agricultural land and crops. Especially in urbanized areas, they are increasing in abundance and are considered nuisance animals. We estimated the population size of carrion crows over the course of one year in relation to waste and non-waste sites in the federal state Vorarlberg, Austria. The current study showed that several human-related food resources influence the abundance of crows. More crows were observed in survey areas of biogas production and green-waste sites compared to reference sites 3 km distant from waste sites. Continuous hunting activities over the past two decades have not reduced population size. We suggest that the sustainable long-term stabilization and reduction of generalist corvid species populations can only be achieved if anthropogenic food resources are limited.

**Abstract:**

In cities and densely populated areas, several corvid species are considered nuisance animals. In Austria, particularly carrion (*Corvus corone*) and hooded crows (*C. cornix*) are regarded as pests by the general public that frequently cause damage to crops, feed on human waste, and thus spread trash. We conducted a detailed one-year field survey to estimate the abundance of carrion crows in relation to potential anthropogenic food sources and reference sites in the Austrian Rhine valley. Our results demonstrated that the number and proximity of waste management facilities, animal feeding areas, and agricultural areas, and the productive capacity of agricultural areas, predominantly influenced habitat choice and abundance of carrion crows. In the current study, the probability of observing more than two carrion crows at a survey site decreased with increasing human population density. Moreover, the abundance of crows increased despite a continuous increase in crow hunting kills registered during the past 25 years. Our study suggests a regionally comprehensive waste management plan could serve as a promising strategy to manage nuisance birds. A reduction in anthropogenic food supply through improved waste management practices is required for long-term, sustainable management to limit the abundance of crow populations in and close to urban environments.

## 1. Introduction

Several bird species adapted to human settlement have increased their abundance in urbanized areas throughout the world. Many crows and ravens (corvids) are opportunistic foragers, generalists that successfully colonize urban habitats and congregate near human-related food sources. Corvids benefit from the anthropogenic impact on the environment caused by urban development, and their populations have increased in cities, suburbs, and agricultural areas worldwide [1,2,3,4,5]. Detailed studies on American crows demonstrate that crow populations increase in areas with more anthropogenic resources, reduce home range size, increase reproduction, and use less space for breeding in urban areas [2,3,6,7]. Several corvid species are considered nuisances or pest animals, and are the focus of agricultural, conservation, and legal control programs [8,9,10,11]. In particular, common ravens (*Corvus corax*), carrion crows (*C. corone*), hooded crows (*C. cornix*), and rooks (*C. frugilegus*) are held responsible for damage to agricultural property and crops, as they break open silage bales [12,13,14] and feed on newly planted seeds in fields [12,15,16,17]. Members of the genus *Corvus* also successfully use waste disposal sites as a reliable food source. An increased abundance of crows is often related to the supplementary food supply [6,18,19,20]. Limiting the amount of, and accessibility to, available waste has been suggested as an effective long-term method to reduce the population of common ravens [21]. Similar suggestions to reduce inadvertently provided food via garbage incineration and dumpster covering have been proposed by the U.S. Fish and Wildlife Service [21,22]. However, regulations and improvements of garbage and waste-disposal management can only take effect when implemented over large geographical areas [21,23,24].

In Austria, waste-disposal management underwent substantial changes due to both the modifications of waste separation and residual waste treatment according to regulations of the European Union and a federal law passed in 2004. The legally regulated threshold required the mechanic, biological, and/or thermal treatment of residual waste instead of the mere disposal of waste. Such treatment is not available in the Austrian federal state of Vorarlberg. Hence, the three main waste disposal sites, which used to deposit garbage without treatment, could no longer be used as such. Waste management in Vorarlberg now depends on the capacity of neighboring provinces or companies in bordering Switzerland. Today, waste is collected and sorted at transfer stations in Vorarlberg and successively relocated for processing. At the current transfer stations, less waste is generally available for crows than in former disposal sites (Markus Boeckle, pers. obs.). However, it remains questionable whether there is a decreased amount of waste available to corvids as a result of this change in waste handling. Additionally, so-called green-waste areas were developed in every community in Vorarlberg to mitigate the deposition of green waste in the countryside. Likewise, biogas production sites in Vorarlberg, which are anaerobic digester facilities that treat farm waste, currently deposit agricultural waste for a short period without covering the to-be-treated waste. All of the above-mentioned waste management areas provide human-related food sources to crows and other corvid species, and thereby might contribute to the increased occurrence and abundance of crows in the vicinity of waste facilities.

In addition to changes in waste management regulations, hunting could potentially negatively impact the abundance of corvid species in general and crows in particular in Vorarlberg. In Austria, hunting is used as a control method to limit the population size of corvids. Since 2009, the European parliament has prohibited the hunting of passerine bird species to conserve wild birds. However, the 1979 adopted and 2009 amended European directive on the conservation of wild birds [16] grants exemptions for carrion crows (*C. corone*), rooks (*C. frugilegus*), western jackdaws (*C. monedula*), Eurasian jays (*Garrulus glandarius*), and Eurasian magpies (*Pica pica*) for several member states, including Austria. In Austria, the game law is regulated by province (Länder) authority, and therefore different regulations apply in different federal provinces. For example, the hunting regulations in the province Burgenland stipulate year-round protection, while in Styria, the hunting of 13,300 hooded crows and 3700 carrion crows per year is allowed, and in Vorarlberg corvid species, among others, are excluded from the conservation of wild living bird species [25]. Several studies, however, report a lack of scientific evidence for a reduction of corvid populations as a result of hunting [26,27,28]. In several cities, populations of carrion crows, hooded crows, and rooks might have increased due to the hunting pressure they experience in the countryside [1,28,29].

Crow breeding pairs, which mainly eat insects that provide sufficient protein for growing juveniles [29], defend food sources and territories against other intruding birds. A reliable availability of subsidized food can increase the breeding performance, including the survival rate of juveniles [1,2]. The size of non-breeder flocks, formed in several corvid species by juveniles and adults alike [30,31,32,33], might increase with food availability in early life stage periods. Additionally, non-breeders often inform each other about the location of food sources using food calls [1,14,34,35,36], and thus a large number of individuals can be recruited to food sources [14,34,37,38,39]. The similar attraction of large numbers of non-breeding individuals gathering at roosts and around substantial but ephemeral food sources is reported for carrion crows [13,15]. It appears likely that subsidized anthropogenic food sources, e.g., waste disposal sites, contribute to an increasing abundance of crows.

The aim of this study was to investigate the effects of anthropogenic food sources on the abundance of carrion crows. Surveys were conducted to examine the spatial relationship between crow abundance and the location of biogas, green-waste sites, and transfer stations, as well as agricultural areas, animal feeding areas, rivers, and the Lake Constance in the Vorarlberger Rhine Valley (federal state Vorarlberg, Austria). Crow abundance and occurrence were recorded with regard to seasonal and daily temporal differences. To evaluate the relationship and effectiveness of recent regulations potentially influencing the population growth of crows, we compared hunting kills and population size reported for corvid species from the past 20 years. We discuss the results of the occurrence and abundance of crows in relation to their ecology and social behavior and in light of current waste-management.

## 2. Materials and Methods

### 2.1. Ethics Statement

The study was approved by the regional environmental office of Vorarlberg. The survey was performed without physical contact with the study animals and did not access privately owned or protected land. The protocol for data collection adhered to the Animal Behavior Society guidelines, and no permit was necessary for the described field observations.

### 2.2. Study Species and Site

Carrion crows have a mean body length of 44–51 cm and wingspan of 84–100 cm; the body mass of females and males ranges from 430 to 650 g [40]. They exhibit a completely black plumage and are closely related to the partly gray hooded crow [41]. The hooded crow is considered a ‘semispecies’ of the carrion crow, as gene flow occurs, but a reduced fitness of hybrids has been reported [42]. In Middle and Southern Europe, *C. corone* breeds predominantly in cultivated, agricultural landscape, along forest edges, in parks, and in marsh areas [17,41]. In the breeding period from March to July, two to seven eggs are laid, and the hatching period lasts up to 22 days [41]. Juveniles are raised by breeding pairs until the summer, and afterwards they are expelled from the natal territory and form non-breeder flocks [41]. Crows are adaptable and opportunistic species, especially regarding their food resources [41]. During winter, crows mostly feed on vegetables, whereas in the summer their diet consists of insects, snails, earthworms, small mammals, bird eggs and fledglings, as well as garbage from waste disposal sites [41,43].

**Figure 1:**
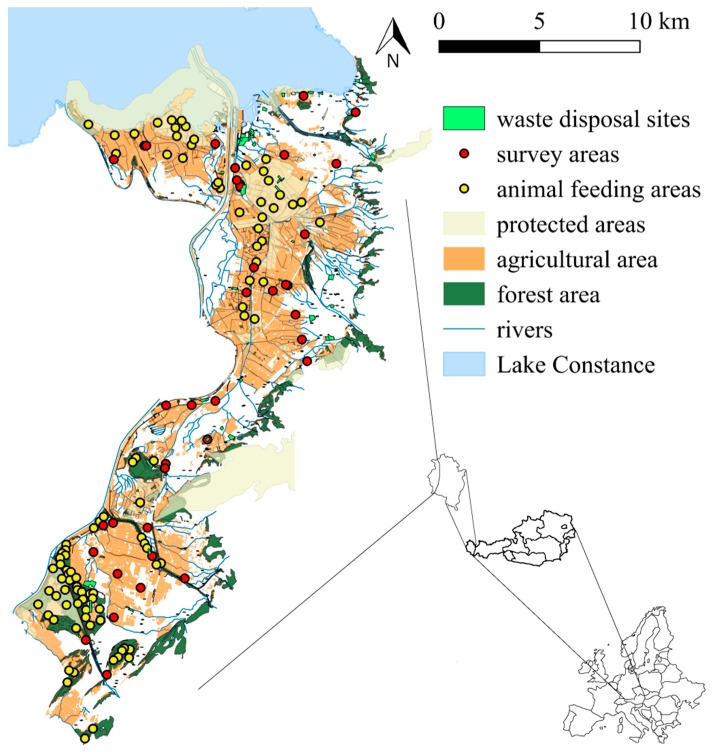
Map of spatial factors that may influence the abundance of *Corvus corone* and *C. cornix* Geographic data included in the full model for the study area are presented including survey areas, waste disposal sites, animal feeding areas, protected areas, agricultural areas, forest areas, and rivers. Maps were provided by http://vogis.cnv.at (© Land Vorarlberg).

The study area is the Austrian Rhine Valley (Rheintal), located in the western part of Austria near the border with Switzerland, 400–500 m above sea level (Figure 1). The area comprises the districts and cities of Feldkirch (34,012 inhabitants), Dornbirn (49,620 inhabitants), and Bregenz (29,826 inhabitants), with an overall estimated population of 395,012 people recorded in December 2018. The valley itself is divided into the upper and the lower part (Oberes Rheintal Unteres Rheintal). The lower part is situated next to the coast of Lake Constance; it comprises about 180,000 inhabitants and hosts about half of the whole federal state of Vorarlberg. Although Vorarlberg is the second smallest state of Austria and has the smallest population of all federal states, it has the second highest population density in the country with 150 inhabitants/km^2^. Most inhabitants live in small towns, which form a long continuous settlement structure including 29 single municipalities. Landscapes between the conurbations are often protected areas under agricultural use. Main forms of agriculture are meadow orchards, pastures for animal husbandry and milk production, croplands for grain (e.g.,: *Triticum aestivum* subsp. *spelta*) and rapeseed (*Brassica napus*), and wood and forestry industries. Lake Constance (47°35′ N, 9°28′ E) is the third largest lake in Europe, covering 571.5 km^2^, and is approximately 395 m above sea level. The 28 km-long coastline of Lake Constance belongs to Vorarlberg, while the remaining coastline runs though Switzerland and Germany. One major highway runs through the valley connecting three major cities of the area and the coastline of Lake Constance with the Arlberg, a mountain range between Vorarlberg and Tyrol. The Rhine Valley is the warmest area of Vorarlberg with a yearly mean temperature between 8 and 9 °C and a yearly rainfall of about 1100 mm.

### 2.3. Data Collection and Analysis

We surveyed the population size of carrion crows for one week and repeated this survey five times within one year. We surveyed 42 selected waste and non-waste sites in week 30 (July) and 40 (October) 2013 and in weeks 5 (January), 19 (May), and 33 (August) 2014, to analyze seasonal patterns of abundance. Survey areas of waste sites included biogas production sites with (*n* = 10) or without (*n* = 5) agriculture, green-waste sites (*n* = 14), wastewater treatment plants (*n* = 3), and transfer stations (*n* = 5). As points of reference in non-waste related sites, we selected reference sites at least 3 km from known waste survey sites in the Rhine Valley. We visited the sites twice a week along a fixed route, with the starting point switched between successive survey occasions. All event sampling was performed for a period of five minutes at the survey sites. At every survey point within those five minutes, we recorded every individual of *Corvus corone* and *C. cornix* seen with the unaided eye within a distance of 300 m. Additionally, we recorded every individual seen within 1 km while we were driving from one site to the next in order to identify areas of high crow abundance other than the focal study sites.

We included information on distance to number of waste deposit sites (all existing deposit sites within the study area) and animal feeding areas (locations with supplementary food supplies from hunters for wild animals, e.g., deer (*Cervus elaphus*, *Capreolus capreolus*), pheasants (*Phasianus colchicus*)) as possible predictor variables in the analysis, as they are potential feeding areas for crows. Further predictor variables were distance to rivers, agricultural areas, protected (conservation-based) areas, and Lake Constance. These distances were included, as they potentially can influence habitat use by crows; distance to Lake Constance is highly related to altitude in the valley. The habitat structure that is close to Lake Constance shows more open areas in agricultural and protected wild life areas, while the habitat progressively changes with increasing distance from the lake to smaller structured and more mountainous habitats. Furthermore, especially in summer, higher temperatures could occur and thus provide potentially better roosting sites. Additionally, we used the capacity value of farmland as a predictor in the models, which was used as a proxy for the productive capacity of an agricultural area—hence the crop yield and/or agricultural output (for the rest of the article abbreviated under the term “capacity value”), which might directly relate to feeding opportunities. Capacity values are evaluated according to economic factors under consideration of environmental influences on a scale of 1–100. All spatial and geographical data used in this study are available from the land surveying office of Vorarlberg VoGis [44]. For calculating geographical and spatial attributes of the survey points, we used the software “R” (R Core Team) [45] and the packages “sp” (version 1.0-16) [46,47]), “rgdal“, (version 0.9-1) [48], “rgeos“ (version 0.3-8) [18], and “geosphere“ (version 1.3-11) [49]. For calculating spatial auto-correlation, we used the package “spdep” (version 1.1-2) [50]. We calculated Moran’s I as a measure of spatial autocorrelation [51] using the package “ape” (version 5.3) [52]. All available shapefiles were converted to the geodetic reference system WGS 84 to build a uniform and standardized analysis basis. The measured data that were assigned to spatial and geographic characteristics and/or distances to geographic elements (e.g., waste related sites, coast of Lake Constance) were calculated. As maximum distance to the next location we estimated 2.5 km, as this will provide distances for all analyzed and calculated variables. The full model included the predictor variables: capacity value, number of waste sites and animal feeding sites within 2.5 km, distance to animal feeding areas and to Lake Constance, daytime (transformed in decimal minutes) and season (winter, spring, summer, and fall), as well as the variable accounting for spatial auto-correlation. We excluded any predictor with a variance inflation factor above 4 in the full model. We specifically excluded human population density, as it showed a high inverse relationship with agricultural usage as measured by capacity value as well as distance to nearest agricultural area as it showed a high relationship with capacity value. In all models, we specified Poisson distribution for the error structure and excluded all interactions between predictor variables. We computed all models using the “R” package “MuMIn” (version 1.15.6) [53] as well as “lme4” (version 1.1-21) [54]. Variance inflation factor was calculated using the package “car” (version 3.0-2) [55]. We selected the best model based on Akaike’s Information Criterion corrected for low sample size (AICc) as the model with the lowest AICc value [56]. We calculated all possible models and ranked them according to delta AICc. We selected the models for which AICc delta was below 6, and calculated model-averaged parameter estimates, ranking them based on how frequently they occured in the previously selected models with delta AICc below 6 [56]. We used Bonferroni correction for multiple comparisons in all models except in the previously described averaged model.

To test the hypothesis that abundance (number of individuals) of crows is increased in areas of biogas, green-waste sites, and transfer stations, we compared 866 GPS logged observations using a Generalized Linear Model (GLM) in Model 1. The number of individuals observed was entered as a dependent variable, with the data on the survey points as predictor variables and the identity number of the survey points as random factor to account for repeated measurements.

In Model 2, a Generalized Linear Mixed Model (GLMM) was performed to analyze if the abundance of crows showed differences based on differences between survey point, daytime, or season at the 42 survey points. The number of individuals observed was entered as dependent variables, with survey point, daytime, and season as predictor variables and the identity number of the survey points as random factor to account for repeated measurements.

The impact of hunting on the abundance of *C. corone*, *C. cornix*, *P. pica*, and *G. glandarius* was documented by comparing the hunting kills from official records for annual hunting seasons in Vorarlberg (http://www.vorarlberg.at/pdf/wildabschussentwicklungab.pdf) with the counts of the respective species from the Bodensee–Brutvogelatlas 2000 [57]. We calculated the percentage of increase or decrease in the number of individuals per species occurring in Vorarlberg reported over a period of 20 years.

Data points were logged with the program GPS Tours on iPhone 4S and the parameters date, taxon, time, GPS coordinate, location, accuracy, and individual number. The resulting data were analyzed with the software “R” (R 3.0.2 GUI 1.62 Snow Leopard build (6558)) and IBM SPSS 19 (IBM, Armonk, NY, USA) for generalized linear mixed models (GLMM) of geographic data.

## 3. Results

During the survey, we recorded 8323 individuals of *Corvus corone* and *C. cornix* at 866 survey points. No crows were observed at 67 survey points. The best model explaining the abundance of crows (*C. corone* and *C. cornix*) included capacity value, distance to the nearest waste disposal site, the number of waste disposal sites within 2.5 km, distance to animal feeding areas, the number of animal feeding areas within 2.5 km, distance to the nearest agricultural area, river, Lake Constance, and protected area, and excluded the distance to the nearest animal feeding area, daytime, and season (Table 1).

In the averaged model, on average two crows were observed (y-intercept = 1.92; GLM: *z*_863_ = 27.5; see Table 2). In agricultural areas with high capacity value, an increased abundance of crows was observed (GLM: *z*_863_ = 46.7; see Table 2). Decreasing number of waste sites within 2.5 km was correlated with crow abundance (GLM: *z*_863_ = −14.2; see Table 2). Similarly, decreasing distance to animal feeding areas (GLM: *z*_863_ = −4.7; see Table 2) increased the abundance of crows, and the number of animal feeding areas predicted higher abundance (GLM: *z*_863_ = 11.0; see Table 2). No significant influence of the distance to Lake Constance (GLM: *z*_863_ = −0.3; see Table 2) and daytime (GLM: *z*_863_ = 0.5; see Table 2) was found. Fall (GLM: *z*_863_ = 8.9; see Table 2) and spring (GLM: *z*_863_ = 19.1; see Table 2) showed lower abundances of crows than summer, while winter had a higher abundance of crows than summer (GLM: *z*_863_ = 8.3; see Table 2). For detailed results, see Table 2.

In Model 2, the abundance of crows in areas of biogas-, green-waste sites, and transfer stations differed significantly from reference sites (GLMM: *F*_42,301_ = 5.499; *p* > 0.001). We found an increased abundance (estimated mean > 10) of individuals of *C. corone* and *C. cornix* at seven waste related sites (Table 3).

**Figure 2:**
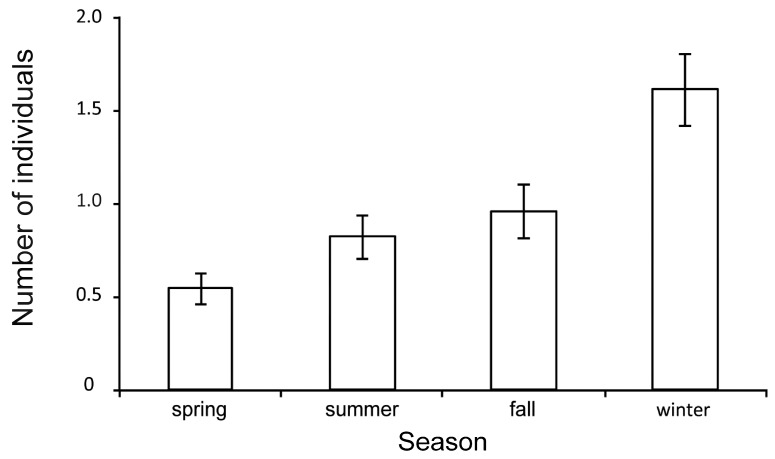
Estimated mean number (±SE) of *Corvus corone* and *C. cornix* observed in four seasons in the federal state of Vorarlberg, Austria.

Crow abundance did not differ in daytime in Model 2 (GLMM: *F*_1, 337_ = 9.639; *p* < 0.05). Seasonal differences in the abundance of *C. corone and C. cornix* were recorded (GLMM: *F*_3, 301_ = 1.245; *p* = 0.265) at the survey points (Figure 2). Abundance was higher in winter compared to spring (GLMM: pair-wise comparison, *ß* = 1.064; SE = 0.206; t = 5.156, *p* = 0.006), summer (GLMM: pair-wise comparison, *ß* = 0.787; SE = 0.224; t = 3.505; *p* = 0.006), and fall (GLMM: pair-wise comparison, *ß* = 0.652; SE = 0.235; t = 2.772; *p* = 0.04). In fall, tendentiously fewer individuals were observed at survey points compared to summer (GLMM: pair-wise comparison, *ß* = −0.413; SE = 0.167; t = −2.476; *p* = 0.084), while no difference in crow abundance was recorded between summer and fall (GLMM: pair-wise comparison, *ß* = −0.135; SE = 0.186; t = −0.729; *p* = 1) as well as spring and summer (GLMM: pair-wise comparison, *ß* = −0.277; SE = 0.139; t = −1.995; *p* = 0.282) (Figure 2).

**Figure 3:**
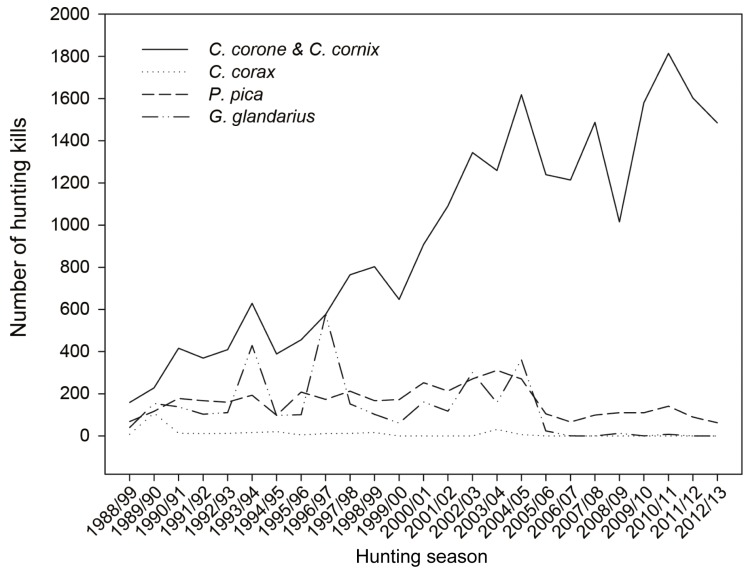
Hunting kills of corvid species (*Corvus corone*, *C. cornix*, *C. corax*, *Pica pica*, and *Garrulus glandarius*) during the annual hunting seasons from 1988 to 2013 in Vorarlberg, Austria.

Crow hunting kills increased during the last 25 years (Figure 3). In the years 1988 to 1990, 2.6 times (161%) more crow hunting kills were registered.

**Figure 4:**
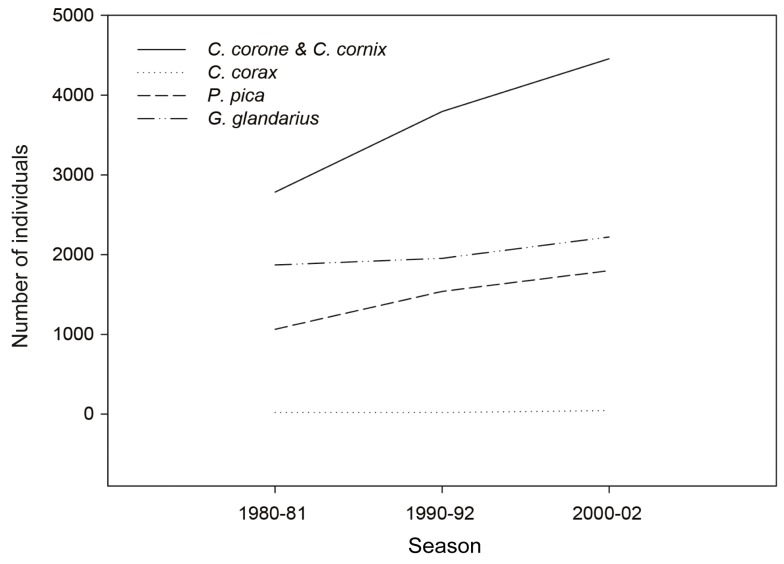
Changes in population sizes of corvid species (*Corvus corone*, *C. cornix*, *C. corax*, *Pica pica*, and *Garrulus glandarius*). Census results from the years 1980–1981, 1990–1992, and 2000–2002. Graphical representations developed from data of the “Brutvogelatlas” [57]. Data are not necessarily described by linear increase but are presented as lines in order to enhance their visibility.

The decennial development from 1990 to 2000 demonstrated a further increase of 118% and a rising trend in the following years [57]. The population size in the years 1980–1982, 1990–1992, and 2000–2002 denoted an increase in the number of individuals (Figure 4).

In 2000, the carrion crow population was 1.6 times higher than in 1980, with an increase of 36% in the first decade and further 17% in the second decade. Carrion crows increased from 2784 individuals in 1980 to 3796 individuals in the year 1990 and up to 4456 in 2000–2002 [57]. Population changes of carrion crows in Austria between 1998 and 2016 report a stable long-term trend without statistically significant variation [58].

## 4. Discussion

The number of recorded crows increased closer to animal feeding areas and Lake Constance. Similarly, higher crow abundance was found in areas with higher agricultural capacity values and more waste- and animal feeding sites. Our findings demonstrate that the abundance of crows increased in developed areas with anthropogenic food sources. Although the best model may not predict the absolute number of crows, it clearly exhibited the relative importance of anthropogenic food sources. Crow abundance was particularly high in areas with supplementary or easily accessible food sources in or close to human settlements.

Our findings agree with those of other studies reporting local increases in crow population size in areas with frequent human activity, food sources, water, and nest site availability, e.g., in common ravens [21,24]. A spatial correlation between the abundance of corvid species and waste disposal sites has also been observed in other provinces of Austria [59]. In the capital Vienna, the number of wintering rooks counted at roosting sites increased by more than 50% from 1992/93 to 1994/95. A large-scale waste disposal site provided food for the most dense roosting site that contained approximately 100,000 rooks [60]. In a further census of rook’s wintering grounds in 1997, 188,719 individuals were counted, representing a doubling of the population size within 10 years [61]. Nevertheless, increases in overwintering populations in cities might result from immigration of birds from more northern and eastern populations (Poland, Finland, Belarus, etc.). Thus, the increase in the number of wintering birds is likely to be related to high productivity during the breeding season in more northern and eastern areas.

The higher abundance of crows close to waste management areas was confirmed in our comparison of waste and non-waste survey points. The seven areas with the highest numbers of crows were in locations of biogas sites, green-waste disposal sites, and transfer stations with uncovered waste that provided a permanent food supply to birds. Predicted abundances from the model with geographical data showed increased abundance of crows when more waste sites are within 2.5 km. This effect might be underestimated because of the inclusion of various waste disposal sites, including disposal sites with no potential food items. The comparison of waste and non-waste sites, however, included only food-related deposit sites and thereby demonstrated that the accessibility of waste in urban areas was directly linked to a higher abundance of crows when comparing waste to non-waste survey points. Our results correspond to recent findings in non-breeders of common ravens deployed with GPS loggers. Analysis of spatial and temporal GPS data showed that ravens spend 75% of the time in close vicinity to anthropogenic food sources [62]. As generalist omnivore, dietary shifts or the exploitation of available food sources can occur rapidly due to the adaptability of corvids to novel resources [2,63].

Waste management changes from former waste disposal sites to transfer stations in Vorarlberg, according regulations of the European Union, were developed to process garbage economically and ecologically and to prohibit pollution of soil and rivers. The alterations in waste handling did not target the management of anthropogenic food sources for birds or other animals. The transformation from waste disposal to transfer stations did seemingly not influence the abundance of carrion crows in the area. Although the amount of accessible waste at transfer stations is less than at former waste disposal sites (MB, pers. obs.), waste, or rather food, remains available for crows, uncovered and thereby easily accessible. Comprehensive, city-wide waste management in Berlin and the closure of the last disposal area in 2005 resulted in a distinct reduction of rooks, hooded crows, and Western jackdaws (*C. monedula*) [64]. Waste management in Berlin has set an example of how an area can prohibit the incentive of additional food resources for corvid species. The implementation of only closed waste treatment facilities reduced the native breeding population of *C. corone* and the migration of *C. frugilegus* [64].

An increased crow abundance was also related to higher capacity value of agricultural land (e.g., higher production of crops), as well as distance to, and number of, agricultural areas. Since human population density is inversely related to agriculture land use, this result demonstrated that increased crow abundance is not restricted to human areas and several anthropogenic food sources facilitate colonization. The majority of protected areas in the Vorarlberger Rhine valley were established to protect the biodiversity of the respective region. Nevertheless, these areas are often used intensively for agriculture, with sufficiently high capacity values or crop production; similarly to areas where animal feeding takes place, these locations provide accessible food sources for crows.

Crow abundance did not increase in the proximity to Lake Constance. The Vorarlberger Rhine Valley is a plain located at low altitude surrounded by high mountains. Hence, our results do not predict that crows are observed at higher abundance in the vicinity of Lake Constance. Still, carrion crows prefer areas up to 1500 m above sea level [65]. The possible influence of low altitude water bodies and surrounding mountains on the abundance of carrion crows suggests that habitat composition can act as a restricting factor for habitat use. This effect, however, was not further investigated in this study, as no observations were made along a wide range of elevations.

On average, two crows could be observed at each observation point in the area of the Vorarlberger Rhine Valley. This observation corresponds to the social structure of territorial corvid species, which occupy territories in pairs [41,43,66]. Seasonal abundance differences were consistent with the behavior of territorial crows. In winter, defense of territorial boundaries by carrion crows is diminished or absent [41,43] thereby allowing a higher abundance of crows [67]. We hypothesize that areas defended by territorial pairs are mainly used by two individuals, while areas that are not defended by a territorial pair are potentially overtaken by a larger number of non-breeding individuals, as suggested by data on ravens [29,68]. Non-breeding flocks form during the winter period, but the individual density is limited by the abundance of food sources [69] unless accessory food or foraging sites are available. Additionally, an increase in the number of individuals within non-breeder flocks might be based on migratory birds joining juvenile and subadult non-breeder flocks (also see [67]). Territories are formed in early spring before the breeding period [41,43,66,69], and crows are less dependent on additional anthropogenic food sources [3], which corresponds to the seasonal observations in our study that showed a decreased abundance during spring. The availability of natural food is presumably higher during this period and not restricted to anthropogenic food sources.

The comparison of population growth and hunting efforts suggests that continuous hunting activities over the past two decades have not achieved the expected reduction in population size of *C. corone* and *C. cornix*. Although population increase might have been slowed as a result, hunting of corvids has been questioned as ineffective and not sustainable [29]. Monitoring of the correct use of granted derogations in order to control corvid populations was advised [70]. The infectivity of a population decrease by hunting might be based on either (i) a high rate of population increase (possibly based on surplus of food availability), which is only slightly curbed by hunting; (ii) most hunting occurring in winter, when many non-resident birds are present, thereby having little impact on the resident local population; or (iii) population growth also resulting from the predominant hunting-kills of territorial breeding pairs. Breeding pairs can be detected in particular territories and thus are more easily located. Similar to common ravens, crows occupy large areas and defend their territory together against competing non-breeding opponents [66], thereby displaying intraspecific spatial avoidance [68,71]. However, if a pair-partner is killed the other partner also leaves the territory [72]. We presume that consequently these territories will be colonized by a larger number of non-breeders, as observed also in common ravens [29]. We suggest the hunting of territorial pairs in addition to anthropogenic food sources promotes the population increase of crows [29]. Accordingly, we assume that due to the abundance of fewer breeding pairs, intraspecific competition for territories and food is reduced. Thus, fewer breeding pairs are able to successfully breed or use more nesting attempts during one year due to subsidized food and additional resources, which in turn can increase the survival rate of juveniles [17,69,73,74]. This leads to a possible increase of number of individuals within the non-breeder flock. Furthermore, large areas without suitable habitat (e.g., agricultural land), as found in our survey areas, offer little or no nesting sites, additionally constrain territorial breeding pairs, and may increase the number of individuals in non-breeder flocks [20,69]. We suggest the constant population growth originates from the vast anthropogenic food resources, and a sustainable effect of hunting remains questionable even in seasons of high hunting returns [73].

Admittedly, the interpretation of our data is limited as our corvid census data collection took place over a single year and research detailing movement and dispersal patterns are needed. The size of the population under investigation might also change across years according to weather fluctuations, agricultural production, etc. The found characteristics might be specific to the observed time period; however, urbanization of highly adaptive corvids is a global phenomenon. Several corvids utilize large home ranges, including a variety of habitats as well as diverse food resources [60,75,76]. We suggest that relatively simple waste control measures could decrease food sources and provide an un-invasive method to limit corvid abundance in urban areas. We assume equal detectability between sites and types of sites, which might influence the recorded data and our results but was used as an approximation. Although the measuring points along a fixed route were travelled several times, each observation was tracked exactly with a GPS. That is to say, there are single points in their proximity we surveyed several times. However, there are also numerous points at which only one survey has been carried out. Therefore, we have not used a repeated measurements account with potential influences on the calculation of variance.

## 5. Conclusions

While most observations focus on the abundance of corvids in cities, the current study showed that human activity and anthropogenic food sources influence the abundance of crows. Agricultural areas; their capacity value; uncovered waste sites; and animal feeding areas, in particular, increase the abundance of crows. Even increased hunting efforts have seemingly had no influence on crow population size in recent years [70]. Following the results of the current and previous studies, we suggest that the sustainable long-term reduction of generalist bird species like *Corvus corone* and *C. cornix* can only be achieved if anthropogenic food sources are limited [64,77]. The current study analyzed for the first time the relationship between anthropogenic food availability and the abundance of crows in Vorarlberg and provides a foundation for management recommendations. Continued studies and surveys would help to identify factors influencing the long-term pattern of population change, as well as effective strategies to reduce crow abundance in human settlements.

## Figures and Tables

**Table 1:** Models with delta AICc below 6.

Model	Intercept	Predictors								df	logLik	AICc	delta	weight
220	1944	1/			4/		6/	7/	8/	9	−9875.68	19,769.60	0.00	0.43
224	1878	1/		3/	4/		6/	7/	8/	10	−9875.17	19,770.60	1.03	0.26
252	1949	1/			4/	5/	6/	7/	8/	10	−9875.42	19,771.10	1.54	0.20
256	1886	1/	2/	3/	4/	5/	6/	7/	8/	11	−9874.97	19,772.20	2.68	0.11

Term Codes: 1: capacity value; 2: season; 3: daytime; 4: distance to nearest animal feeding area; 5: distance to Lake Constance; 6: number of waste disposal sites within 2.5 km; 7: number of animal feeding areas within 2.5 km; and 8: auto-covariation. Model 256 represents the full model. df: degrees of freedom; LogLik: logistical likelihood; AICc: corrected Akaike Information Criterion; delta: difference of AICc between the models; weight: model weight.

**Table 2:** Summary results of crow abundance after model averaging: effects of each parameter on crow abundance.

Parameter	Estimate	Unconditional SE	CI	Relative Importance
(Intercept)	1.921	0.070	(1.784, 2.05)	
Capacity value	0.001	0.000	(0.00081, 0.00089)	1.00
Season 2 *	−0.279	0.031	(−0.340, −0.218)	1.00
Season 3	0.229	0.027	(0.175, 0.283)	1.00
Season 4	−0.680	0.035	(−0.750, −0.611)	1.00
Distance to feeding area	0.00004	0.000	(0.000024, 0.000059)	1.0
Auto-covariation	0.006	0.002	(0.0020, 0.0094)	1.0
Number or waste disposal sites	−0.011	0.001	(−0.0125, −0.0095)	1.0
Number of animal feeding sites	0.021	0.002	(0.018, 0.025)	1.0
Daytime	0.00003	0.000	(−0.000088, 0.000268)	0.37
Distance to lake of Constance	−0.0000003	0.000	(−0.0000034, 0.0000017)	0.31

* Season 1 was the reference category; summer = 1, fall = 2, winter = 3, and spring = 4.

**Table 3:** Survey sites with high abundance of crows in the Vorarlberger Rhine Valley, Austria. All survey points where the mean number of crows observed (*Corvus corone* and *C. cornix*) exceeded 10 are listed. Values represent estimates from generalized linear model analysis.

Survey Point Number/Description	Number of Crows/Site			
	Mean	SE	Minimum	Maximum
ID4/Biogas	19.0	6.7	9.5	37.9
ID9/Biogas	88.2	27.6	47.7	163.2
ID11/Biogas	14.1	5.4	6.6	29.9
ID26/Biogas	56.3	15.0	33.3	95.1
ID27/Transfer station	46.6	16.0	23.7	91.5
ID29/Green waste	16.8	5.4	9.0	31.5
ID32/Biogas	15.4	5.3	7.8	30.5
